# Body Mass Index Is Inversely Associated with Risk of Postmenopausal Interval Breast Cancer: Results from the Women’s Health Initiative

**DOI:** 10.3390/cancers14133228

**Published:** 2022-06-30

**Authors:** Zhenzhen Zhang, Grace Curran, Jackilen Shannon, Ellen M. Velie, Veronica L. Irvin, JoAnn E. Manson, Michael S. Simon, Duygu Altinok Dindar, Chelsea Pyle, Pepper Schedin, Fred K. Tabung

**Affiliations:** 1 Division of Oncological Sciences, Oregon Health & Science University, Portland, OR 97239, USA; shannoja@ohsu.edu; 2 Knight Cancer Institute, Oregon Health & Science University, Portland, OR 97239, USA; currangr@ohsu.edu (G.C.); altinokd@ohsu.edu (D.A.D.); schedin@ohsu.edu (P.S.); 3 Cancer Early Detection Advanced Research Center, Oregon Health & Science University, Portland, OR 97239, USA; 4 Zilber School of Public Health, University of Wisconsin-Milwaukee, Milwaukee, WI 53205, USA; velie@uwm.edu; 5 Departments of Medicine and Pathology, Medical College of Wisconsin, Milwaukee, WI 53226, USA; 6 College of Public Health and Human Sciences, Oregon State University, Corvallis, OR 97330, USA; veronica.irvin@oregonstate.edu; 7 Department of Medicine, Brigham and Women’s Hospital, Harvard Medical School, Boston, MA 02115, USA; jmanson@rics.bwh.harvard.edu; 8 Department of Epidemiology, Harvard T.H. Chan School of Public Health, Boston, MA 02115, USA; 9 Karmanos Cancer Institute, Department of Oncology, Wayne State University, Detroit, MI 48202, USA; simonm@karmanos.org; 10 Department of Radiology, Oregon Health & Science University, 3181 SW Sam Jackson Park Road, Portland, OR 97239, USA; pylec@ohsu.edu; 11 Department of Cell, Developmental and Cancer Biology, Oregon Health & Science University, Portland, OR 97239, USA; 12 Department of Internal Medicine, Division of Medical Oncology, College of Medicine and Comprehensive Cancer Center, The Ohio State University, Columbus, OH 43210, USA; fred.tabung@osumc.edu

**Keywords:** interval breast cancer, BMI, obesity, WHI, breast cancer

## Abstract

**Simple Summary:**

Breast cancer diagnosed between a negative screening mammogram and the next regularly scheduled mammographic exam is called interval breast cancer. It is often diagnosed at more advanced stages than screening-detected cancers. While body mass index (BMI) is a risk factor for postmenopausal breast cancer overall and can influence the accuracy of mammography, the association of BMI with postmenopausal interval breast cancer is unclear. Using data from the Women’s Health Initiative, a national study among postmenopausal women, we found that lower BMI was significantly associated with a higher risk of interval breast cancers diagnosed within 1 year of a negative mammogram after adjustment for multiple risk factors. These findings suggest that obesity is associated with a lower risk of postmenopausal interval breast cancer. Future research using body composition measures is warranted to confirm our findings.

**Abstract:**

Interval breast cancer refers to cancer diagnosed after a negative screening mammogram and before the next scheduled screening mammogram. Interval breast cancer has worse prognosis than screening-detected cancer. Body mass index (BMI) influences the accuracy of mammography and overall postmenopausal breast cancer risk, yet how is obesity associated with postmenopausal interval breast cancer incidence is unclear. The current study included cancer-free postmenopausal women aged 50–79 years at enrollment in the Women’s Health Initiative who were diagnosed with breast cancer during follow-up. Analyses include 324 interval breast cancer cases diagnosed within one year after the participant’s last negative screening mammogram and 1969 screening-detected breast cancer patients. Obesity (BMI ≥ 30 kg/m^2^) was measured at baseline. Associations between obesity and incidence of interval cancer were determined by sequential logistic regression analyses. In multivariable-adjusted models, obesity was inversely associated with interval breast cancer risk [OR (95% CI) = 0.65 (0.46, 0.92)]. The inverse association persisted after excluding women diagnosed within 2 years [OR (95% CI) = 0.60 (0.42, 0.87)] or 4 years [OR (95% CI) = 0.56 (0.37, 0.86)] of enrollment, suggesting consistency of the association regardless of screening practices prior to trial entry. These findings warrant confirmation in studies with body composition measures.

## 1. Introduction

Interval breast cancer refers to cancer emerging after a non-suspicious mammography screen, and prior to the next scheduled screen. Previous studies have found that interval breast cancer has greater clinical severity at diagnosis, including higher average histological grade, larger tumor size, more metastatic local lymph nodes, and a worse prognosis compared to screening-detected cancers [1,2,3,4,5,6]. Women diagnosed with interval breast cancer are also reported to be more likely to have had a prior cancer diagnosis (other than breast cancer) and to be at higher risk for cancers other than breast cancer after an interval breast cancer diagnosis [2], implicating unique exposures and/or family history. Approximately 20–30% of newly diagnosed breast cancers among postmenopausal women attending mammography were interval breast cancers [4,7]. With an estimated 287,850 new cases of female breast cancer in 2022, interval breast cancers are far from rare [8]. An interval breast cancer diagnosis can be due to either true aggressive tumor biology, or ‘masking’ of existing cancer, i.e., a false negative in the last mammographic screen [9]. For example, dense breast tissue may obscure detection of a tumor on mammography, a limitation that could be resolved with ultrasound and MRI [4]. Alternatively, or perhaps in addition, the underlying biology of the tumor may drive more aggressive cancer. Risk factors for interval breast cancer have been largely understudied, and because it is such an aggressive disease, identifying high-risk populations should be prioritized for improved early detection and diagnosis. 

The current study objective was to investigate the role of BMI at enrollment with subsequent interval breast cancer risk using data from the Women’s Health Initiative (WHI) Clinical Trials [10]. A previous study of the WHI clinical trial cohort showed that obesity was associated with overall increased invasive breast cancer risk [11]. Furthermore, postmenopausal breast cancer has been identified more often and at more advanced stages in obese women, and rates of recall and biopsy are also higher in this population [12], as are increased cancer size and stage upon diagnosis [13]. Obese women with dense breast had a 6-fold increased risk for postmenopausal breast cancer compared to underweight women [14]. BMI and associated adipose tissue have been shown to influence underlying tumor biology and risk of breast cancer [15,16] and through similar mechanisms may influence risk of interval breast cancer. Although obesity has been identified as a risk factor for breast cancer in postmenopausal women [17] and a protective factor for breast cancer in premenopausal women [18], how obesity is associated with interval breast cancer is not well documented. 

Obesity and body mass index (BMI) have been shown to influence the accuracy of screening mammograms [19], and thus may contribute to interval breast cancer diagnoses. Obese women with fattier breast tissue have been shown to be 20% more likely to receive false-positive findings from mammograms compared to women of underweight and normal weight [19]. On the contrary, women with BMI < 25 kg/m^2^ are 30% more likely to receive false-negative results compared to those with BMI > 25 kg/m^2^ among postmenopausal women 50–59 years old [20], raising the possibility that low BMI could be a risk factor for interval breast cancer. In addition, breast density and BMI are inversely associated with each other and may act synergistically in breast cancer [14,21]. Lean women have an increased risk of dense breasts, which is a strong risk factor for breast cancer [22], including interval breast cancer [23]. Biologically, mammographic density is associated with breast stroma composition [24] with dense areas especially associated with higher pro-tumor fibrillar collagen deposition [25]. Collagen can directly increase the matrix stiffness and indirectly modulate mammary fibroblast secretion of soluble factors such as transforming growth factor beta, insulin-growth factor, and epidermal growth factor to promote tumorigenesis [24], leading to tumor progression. Determining associations between obesity and interval breast cancer, as performed in this current study, could inform future efforts to understand and target the influence of BMI and associated adipose tissue on the underlying tumor biology and or masking of interval breast cancers. 

## 2. Methods

### 2.1. Study Population

Data were gathered from the Women’s Health Initiative (WHI), a sample of breast cancer-free postmenopausal women (at time of enrollment), ages 50–79 years old (*n* = 161,808) from 1993–1998 with follow-up through mid-2019. For the current analyses, we only included WHI participants (1) diagnosed with breast cancer during follow-up; (2) enrolled in either or both of the WHI clinical trials (Hormone Therapy (HT) Trials and/or the Dietary Modification (DM) Trial); (3) compliant with the protocol-mandated screening guideline. Women were excluded if they were not compliant with the protocol-mandated screening guideline, had contradictory recordings or missing data on key mammogram information, or had interval breast cancer diagnosed 1–2.5 years after their last negative mammogram.

### 2.2. Assessment of Interval Breast Cancer and Screening-Detected Breast Cancer

**Figure 1:**
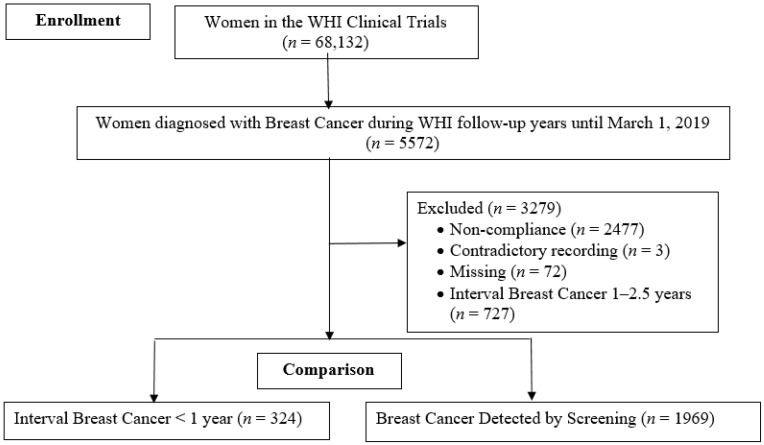
Flow chart of Women’s Health Initiative (WHI) participants included in the analyses.

The primary outcome of the current analyses was interval breast cancer, defined as breast cancer that presented symptomatically after a negative mammographic screen and before the next scheduled mammogram, as compared to screening-detected breast cancer. We identified 1050 interval breast cancer cases (*n* = 324 at <1 year and *n* = 726 at 1–2.5 years after a negative mammogram), and 1969 screening-detected breast cancer cases [1] (see Figure 1). Interval breast cancer cases were identified based on mammogram history, date of last mammogram, type of visit and mammogram exam results. From the WHI data, interval breast cancer was defined with a diagnosis date between the recommended screening intervals of 2.5 years for participants in the DM arm, and 1.5 years for participants in the HT arm. From prior research, interval cancers diagnosed within 1 year of the prior mammogram had characteristics associated with worse prognosis [1], and this group was chosen for the current analysis.

### 2.3. Assessment of Exposures 

Participant height, weight, waist circumference and hip circumference were measured at baseline (ages 50–79 years; mean age = 63 years) by trained interviewers. Body mass index (BMI) was calculated as (weight [kilograms]/height squared [meters squared]) and categorized based on National Heart Lung and Blood categories [26]: underweight BMI, <18.5 kg/m^2^; normal, 18.5 kg/m^2^ to <25 kg/m^2^; overweight, 25 kg/m^2^ to <30 kg/m^2^; obese, ≥30 kg/m^2^. 

### 2.4. Assessment of Covariates 

All covariates were self-reported by participants in the WHI enrollment questionnaires completed between 1993–1998. Covariates considered in this analysis included: age at study entry, highest education, parity, family history of breast cancer, waist-to-hip ratio (WHR), comorbidity, energy expended from recreational physical activity (MET-hr/wk) and current smoking and alcohol use status (Table 1). WHR was calculated by waist circumference divided by hip circumference; it reflects central obesity [27]. Comorbidity at enrollment was calculated with the Charlson comorbidity index [28] based on baseline data reported by the participants. Additionally, participants completed a food frequency for habitual diet assessment, from which total dietary energy intake (kcal/day) was calculated. A Gail 5-year risk score was also calculated at baseline based on age, age of menarche, age at first live birth, history of first-degree relative with breast cancer, history of previous breast biopsy and race/ethnicity.

### 2.5. Statistical Analysis

We first compared anthropometric, reproductive, lifestyle and health behavior characteristics between women diagnosed with interval breast cancer against those with screening-detected breast cancer. Bivariate associations between BMI and each of the studied covariates were analyzed using *t*-tests for continuous variables and Chi-square tests for categorical variables. Continuous BMI analysis was represented as per one-unit increases; categorical BMI was calculated with normal weight (BMI: 18.5–24.6) as reference.

Covariates were selected for inclusion in logistic regression models based on unadjusted analyses including analyses of BMI with multiple variables and IBC risk with multiple variables, as well as risk factors for breast cancer previously identified in the literature [29]. Covariates considered in adjusted analyses included: Gail 5-year risk score, waist-to-hip ratio, total energy intake, total energy expended from recreational physical activity (MET-h/week), hormone replacement therapy clinical trial arm/dietary modification trial arm, smoking status, total alcohol intake, education, and comorbidity. Five sequential multivariable-adjusted models with BMI included as the main exposure of interest were fit, with new variables added to each model. In sequential order, models were (1) unadjusted, (2) WHR, (3) Gail 5-year risk, (4) total energy intake (from diet) and expenditure (from recreational physical activity), and (5) hormone replacement therapy clinical trial arm and dietary modification trial arm, smoking status, alcohol intake, education, and comorbidity. We also conducted another model equivalent for our final model (5) where we replaced Gail 5-year risk score with the original variables comprising the score: age, ethnicity, age at menarche, age at first full term birth, family history of breast cancer, and previous breast biopsy.

We conducted sensitivity analyses to tease out the screening practice impact prior to trial entry by excluding (1) all breast cancer cases diagnosed within 2 years after enrolling into the WHI study and (2) all breast cancer cases diagnosed within 4 years after enrolling into the WHI study. We also conducted stratified analyses by early-stage vs. late-stage breast cancer. The current analytic study participants included those receiving estrogen therapy alone (*n* = 151) and their controls (*n* = 212), and those receiving both estrogen and progestin therapy (*n* = 398) and their controls (*n* = 297). Since the original WHI study found women receiving estrogen and progestin had significantly increased risk of breast cancer [30], we also conducted additional sensitivity analyses by excluding women receiving estrogen and progestin.

## 3. Results

Table 1 shows the unadjusted comparisons of demographic and lifestyle characteristics between participants with interval breast cancer and screening-detected breast cancer. At baseline, slightly fewer women (70.06%) were overweight or obese in the interval breast cancer group compared to the screening-detected breast cancer group (77.30%). We found BMI at enrollment to be inversely associated with interval breast cancer risk in both continuous and categorical models, *p* < 0.0001. To further investigate this potential inverse relationship, we evaluated the association between BMI and sociodemographic, medical history and lifestyle characteristics, including, waist-to-hip ratio at enrollment, height, Gail 5-year risk score, total energy intake and energy expended from recreational physical activity (MET-hrs/wk) (Table 2). Chi-square results showed that BMI was different by race/ethnicity (*p* < 0.0001), age at menarche (*p* < 0.0001) and age at first live birth (*p* < 0.0001), instance of previous breast biopsy (*p* = 0.0004), number of comorbidities (*p* < 0.0001), education level (*p* < 0.0001), alcohol use (*p* < 0.0001) and membership in WHI Hormone Therapy study arms (*p* < 0.0001). Those with a higher BMI were also more likely to be randomly assigned to the hormone intervention group at baseline. We did not find associations between BMI and age at enrollment or diagnosis, family history of breast cancer, parity, or Dietary Modification trial arm membership. 

Table 3 reports the associations between BMI and interval breast cancer with a series of sequential multivariable-adjusted logistic regression models. We conducted the same sequential modeling, treating BMI as a continuous variable with results shown in Table 3. We found women with obesity were at lower risk of interval breast cancer [OR (95% CI) = 0.64 (0.45, 0.91)]. Every one-unit increase in BMI was associated with 4% decreased risk of interval breast cancer in the adjusted models (Table 3). Detailed results for other covariates are presented in Supplementary Tables S1 and S2. To address potential collinearity between WHR and BMI, we used the same adjusted covariates in model 5 with WHR as the main exposure variable without including BMI. Our results showed WHR lost statistical significance with interval breast cancer (Supplemental Table S3). In model 6, we replaced the Gail 5-year risk score with the original components of the Gail model including age, ethnicity, age at menarche, age of the mother at the birth of her first live child, family history of breast cancer, and the number of previous breast biopsy examinations. The results still showed an association between BMI and interval breast cancer with a one unit increase of BMI having 3% reduced risk for interval breast cancer [OR (95% CI) = 0.97 (0.94, 0.99)]. These inverse association results differed from the positive association results on obesity and overall incident breast cancer previously reported in WHI clinical trial cohorts [11].

Table 4 shows sensitivity analyses examining risk of interval breast cancer by BMI, excluding cases diagnosed within less than 2 years or 4 years after enrollment in the study, and by excluding women on an estrogen and progestin trial arm, and by only including early-stage or late-stage breast cancer. Results revealed a consistent inverse association between BMI and interval breast cancer risk.

## 4. Discussion

Our study shows increasing BMI was inversely associated with risk of postmenopausal interval breast cancer, which differs from previously reported positive associations between obesity and overall postmenopausal invasive breast cancer risk [12]. In unadjusted and adjusted analyses, women with interval breast cancer had lower continuous BMI scores on average and a lower proportion were obese compared with their screening-detected peers. Each one-unit increase in BMI was associated with a 4% decreased risk of interval breast cancer in the adjusted model. The results remained statistically significant even after adjustment for WHR, Gail 5-year-risk-score, educational level, clinical trial arm, comorbidities, dietary energy intake, physical activity, and cigarette and alcohol intake. The findings suggest that lower BMI is independently associated with increased risk of postmenopausal interval breast cancer. Waist circumference, which correlates with BMI and reflects central obesity, has been found to be significantly associated with postmenopausal breast cancer [27], yet in our study, the strength of the association between WHR and interval breast cancer lost significance in the adjusted model, although the relationship was inverted, like the relationship between BMI and interval breast cancer. These results suggest BMI is a dominant anthropometric measurement impacting a woman’s risk for interval breast cancer. Sensitivity analyses were conducted to examine this relationship with screening history and with assignment to estrogen or progestin trial arms. Sensitivity analyses revealed a robust inverse association between BMI and interval breast cancer risk. 

Previous studies of premenopausal women with early-onset breast cancer have shown obesity is associated with a reduced risk of breast cancer; that is, the higher the BMI, the lower the risk [17]. A change appears in postmenopausal women, with BMI as a risk factor rather than a protective factor [17]. In younger women, obesity is associated with decreased estrogen activity, possibly via ovarian suppression as estrogens are mainly synthesized in the ovaries [31], whereas in postmenopausal women obesity is associated with increased estrogen activity through the production of aromatase by adipocytes [32]. A previous study using WHI clinical trial data, which included 3388 incident overall breast cancer cases, confirmed the positive association between obesity and overall breast cancer risk [11]. Our previous study [1] suggests that even after adjusting for stage or lymph node involvement and tumor size, interval breast cancer diagnosed less than one years still showed poorer survival compared to screening-detected breast cancer; however, no difference in survival was observed between interval breast cancers diagnosed more than one year and screening-detected breast cancer. Thus, unique biology, rather than simply a delayed diagnosis, contributes to the interval breast cancer rather than delayed diagnosis. One implication of our finding that lower BMI was associated with higher risk of more aggressive interval breast cancer diagnosed within 1 year of negative mammogram results among postmenopausal women is that lean postmenopausal women, who collectively are anticipated to have less estrogen activity, are at increased risk for aggressive postmenopausal interval breast cancer. These observations suggest that interval cancers may be less dependent on estrogen signaling. Since both early-onset breast cancer and interval breast cancer share aggressive tendencies, further studies should compare the two in terms of biological mechanisms. 

Our data are also consistent with interval breast cancer being masked by denser breast tissue found in lean women, a potential interaction that requires further study. Dense breast tissue has a higher proportion of false-negative mammographic results [20]. Premenopausal women generally have denser breasts than postmenopausal women due to ovarian hormones that cause an increase in fibroglandular tissue compared to fat, and thus, more dense breasts [33]. Whether postmenopausal women with interval breast cancer share similar breast density patterns with women with premenopausal breast cancer remains to be determined. Studies have observed a correlation between BMI and breast volume and breast density [34]; both percent density and adiposity are positively associated with breast cancer risk but negatively associated with one another [35]. Our study shows that lean women have a higher risk for interval breast cancer. Therefore, one possible explication for this higher risk is a dominant effect of higher mammographic density among lean women. Alternate methods of breast density measurements have been suggested and studied, including total breast volume and absolute dense volumes, and may provide a more accurate tool for interval breast cancer or screening-detected breast cancer risk [36].

A limitation of our study is that our sample lacked objective measures of body composition to appropriately differentiate between fat mass and fat-free mass and their distribution around the body. We used BMI and waist -hip -ratio (WHR) measurements in the analyses. BMI represents a person’s general (subcutaneous) adiposity [37]; however, it does not differentiate between subcutaneous adiposity, visceral adiposity or muscularity, which have distinct impacts on breast cancer risk [38,39,40]. Previous studies have shown that even among postmenopausal women with a normal BMI range of 18.5 to <25 kg/m^2^, those with a relatively high trunk fat (a potential surrogate for visceral fat) are at significantly elevated risk of invasive breast cancer and show altered concentrations of circulating inflammatory and metabolic factors [41,42]. WHR is a better approximation of fat distribution around the abdominal area than BMI and, if inflammation is a risk factor for interval breast cancer, might be expected to be more strongly correlated with interval breast cancer in our study. However, our earlier work did not find associations between diet-driven inflammation and insulinemia and risk of interval breast cancer [43]; in this study, we did not observe statistically significant associations with interval breast cancer risk when we replaced BMI with WHR as the main exposure. Given that we did not see an improvement in the ability to predict interval breast cancer with WHR, we suggest that the driving relationship between obesity and interval breast cancer may be mainly due to general adiposity and less due to visceral adiposity. Further studies on objective measures of body composition that can differentiate between fat mass and fat-free mass are needed.

Our study has several strengths, including a large population-based cohort with a long -follow -up period of 19 years (median), extensive case ascertainment and mammography history, and collection of detailed information on covariates. We were able to exclude women who were non-compliant with screening recommendations in order to focus only on screen-compliant women. Our study is limited by the fact that we were unable to consider the independence or interaction between breast density and BMI, given that density data were not collected. The results from this study may not be generalizable to non-US population.

## 5. Conclusions

We found that lower BMI was associated with higher risk of postmenopausal interval breast cancer diagnosed <1 year from a normal screening mammogram, when compared with screening-detected breast cancer. Our findings need confirmation in future studies with objective measures of body composition and fat distribution. In addition, there is need for additional research on screening techniques and prediction models beyond mammography. 

## Figures and Tables

**Table 1:** Demographic and lifestyle characteristics of women diagnosed with interval breast cancer and screening-detected breast cancer.

Variable	Interval Breast Cancer (*n* = 324)	Breast Cancer Detected by Screening (*n* = 1969)	*p*-Value
Demographic characteristics			
Age at enrollment, mean (SD), y	63.07 (7.15)	63.24 (6.79)	0.68
BMI (kg/m^2^) at enrollment, mean (SD) continuous variable	28.08 (5.35)	29.46 (5.74)	<0.0001
BMI (kg/m^2^) at enrollment, categorical variable			<0.0001
Underweight	2 (0.62)	8 (0.41)	
Normal weight	95 (29.32)	439 (22.30)	
Overweight	126 (38.89)	675 (34.28)	
Obese	101 (31.17)	847 (43.02)	
Waist-to-hip ratio at enrollment, mean (SD)	0.81 (0.07)	0.82 (0.08)	0.03
Height (cm), mean (SD)	161.8 (6.48)	162.2 (6.49)	0.25
Gail 5-yr risk score, mean (SD)	2.00 (1.22)	1.86 (1.08)	0.03
Race/ethnicity, No. (%)			0.24
White	276 (85.2)	1688 (85.9)	
African American	22 (6.8)	160 (8.1)	
Hispanic	14 (4.3)	44 (2.2)	
Asian	8 (2.5)	45 (2.3)	
Other	4 (1.2)	28 (1.4)	
Missing	0	4	
Family history of breast cancer, No. (%)			0.22
Yes	80 (25.9)	423 (22.7)	
No	229 (74.1)	1441 (77.3)	
Missing	15	105	
Ever full-term birth, No. (%)			0.54
Yes	273 (96.5)	1732 (97.1)	
No	10 (3.5)	51 (2.9)	
Missing	41	186	
Age at first live birth, No. (%)			0.11
Never had any live birth	8 (3.17)	46 (2.87)	
<20 years old	28 (11.11)	269 (16.80)	
20–29 years old	184 (73.02)	1122 (70.08)	
≥30 years old	32 (12.70)	164 (10.24)	
Missing	72	368	
Age at menarche, No. (%)			0.09
≤11 years old	66 (12.38)	467 (23.85)	
12 years old	89 (27.47)	490 (25.03)	
13 years old	110 (33.95)	559 (28.55)	
14 years old	35 (10.80)	288 (14.71)	
≥15 years old	24 (7.41)	154 (7.87)	
Missing	0	11	
Previous breast biopsy, No. (%)			0.09
0	197 (68.17)	1286 (73.53)	
1	65 (22.49)	351 (20.07)	
>1	27 (9.34)	112 (6.40)	
Missing	35	220	
Total dietary energy intake (kcal/day)	1683.8 (650.4)	1733.4 (703.9)	0.24
Total energy expend from recreational physical activity (MET-hours/week)	10.42 (10.60)	9.74 (10.85)	0.29
HT Study group, No. (%)			0.37
Estrogen-alone intervention	20 (15.27)	131 (14.13)	
Estrogen-alone control	21 (16.03)	191 (20.60)	
Estrogen + progestin intervention	57 (43.51)	341 (36.79)	
Estrogen + progestin control	33 (25.19)	264 (28.48)	
Not randomized to HT	193	1042	
HT Study group Re-group, No. (%)			0.95
Estrogen-alone intervention	20 (6.17)	131 (6.65)	
Estrogen + progestin intervention	57 (17.59)	341 (17.32)	
No Estrogen or progestin Intervention	247 (76.23)	1497 (76.03)	
DM Trial group, No. (%)			0.80
Intervention	92 (39.66)	521 (38.79)	
Control	140 (60.34)	822 (61.21)	
Not randomized to DM	92	626	
Comorbidity at Enrollment, No. (%)			0.45
0	222 (68.52)	1364 (69.34)	
1	66 (20.37)	423 (21.50)	
2	23 (7.10)	131 (6.66)	
≥3	13 (4.01)	49 (2.49)	
Education			0.27
Below high school	11 (3.42)	87 (4.47)	
High school diploma/GED	49 (15.22)	331(16.98)	
Vocational or training school	38 (11.80)	215 (11.03)	
Some college or associate degree	87 (27.02)	585 (30.02)	
College degree or baccalaureate degree	46 (14.29)	200 (10.26)	
Postgraduate degree	91 (28.26)	531 (27.24)	
Missing	2	20	
Smoking			0.07
Never smokers	169 (53.14)	977 (50.28)	
Current smokers	28 (8.81)	120 (6.18)	
Ever smokers	121 (38.05)	846 (43.54)	
Missing	6	26	
Alcohol			0.64
Never drinkers	38 (11.91)	221 (11.34)	
<1 drink/month	48 (15.05)	277 (14.22)	
<1 drink/week	63 (19.75)	402 (20.64)	
1– < 7 drinks/week	75 (23.51)	478 (24.54)	
≥7 drinks/week	43 (13.48)	206 (10.57)	
Past drinkers	52 (16.30)	364 (18.69)	
Missing	5	21	

Note: Chi-square tests were used for categorical variables and *t*-tests were used for continuous variables. Missing categories were excluded from statistical analysis. Abbreviations: BMI: Body mass index; HT: Hormone therapy; DM: Dietary modification; SD: Standard deviation; MET: Metabolic equivalent of task.

**Table 2:** Associations between BMI and covariates.

Variable	Normal Weight (BMI: 18.5–24.9) (*n* = 534)	Overweight (BMI: >24.9–29.9) (*n* = 801)	Obese (BMI: >29.9) (*n* = 948)	*p* Value
Continuous variables				
Age at enrollment, mean (SD), y	63.13	63.37	63.16	0.75
Age at diagnosis, mean (SD), y	68.29	68.58	68.60	0.69
Growth morphometric variables, mean (SD)				
Height (cm)	163.38	162.13	161.39	<0.0001
Waist-to-hip ratio at enrollment	0.77	0.82	0.85	<0.0001
Gail 5-yr risk score	1.95	1.92	1.80	0.02
Total energy intake	1603.95	1687.14	1832.18	<0.0001
Total energy expended from recreational physical activity (MET-hours/week)	12.51	10.57	7.72	<0.0001
Categorical variables				*p* value
Race/ethnicity, No. (%)				<0.0001
White	479 (89.70)	707 (88.38)	769 (81.38)	
African American	22 (4.12)	47 (5.88)	113 (11.96)	
Hispanic	8 (1.50)	20 (2.50)	30 (3.17)	
Asian	22 (4.12)	15 (1.88)	16 (1.69)	
Other	3 (0.56)	11 (1.38)	17 (1.80)	
Family history of breast cancer, No. (%)				0.95
Yes	121 (23.68)	176 (23.01)	204 (23.00)	
No	390 (76.32)	589 (76.99)	683 (77.00)	
Ever full-term birth, No. (%)				
Yes	460 (97.05)	703 (97.10)	835 (96.98)	0.99
No	14 (2.95)	21 (2.90)	26 (3.02)	
Age at first live birth, No. (%)				<0.0001
Never had any live birth	13 (2.97)	19 (2.92)	22 (2.90)	
<20 years old	50 (11.42)	86 (13.23)	161 (21.21)	
20–29 years old	326 (74.43)	482 (74.15)	493 (64.95)	
≥30 years old	49 (11.19)	63 (9.69)	83 (10.94)	
Age at menarche, No. (%)				<0.0001
≤11 years old	89 (16.82)	178 (22.33)	265 (28.01)	
12 years old	121 (22.87)	201 (25.22)	255 (26.96)	
13 years old	176 (33.27)	235 (29.49)	253 (26.74)	
14 years old	93 (17.58)	121 (15.18)	107 (11.31)	
≥15 years old	50 (9.45)	62 (7.78)	66 (6.98)	
Previous breast biopsy, No. (%)				0.0004
0	310 (66.67)	506 (72.29)	661 (76.42)	
1	117 (25.16)	134 (19.14)	153 (18.84)	
>1	38 (8.17)	60 (8.57)	41 (4.74)	
HT study group, No. (%)				0.0002
Estrogen-alone intervention	27 (12.74)	36 (10.26)	88 (17.96)	
Estrogen-alone control	29 (13.68)	65 (18.52)	118 (24.08)	
Estrogen + progestin intervention	93 (43.87)	143 (40.74)	159 (32.45)	
Estrogen + progestin control	63 (29.72)	107 (30.48)	125 (25.51)	
Not randomized to HRT	322	450	458	
DM trial group, No. (%)				0.12
Intervention	135 (37.71)	237 (42.17)	238 (36.62)	
Control	223 (62.29)	325 (57.83)	412 (63.38)	
Not randomized to DM	176	239	298	
Comorbidity at enrollment, No. (%)				<0.0001
0	404 (75.66)	576 (71.91)	601 (63.40)	
1	96 (17.98)	160 (19.98)	230 (24.26)	
2	26 (4.87)	50 (6.24)	78 (8.23)	
≥3	8 (1.50)	15 (1.87)	39 (4.11)	
Education				<0.0001
Below high school	9 (1.69)	30 (3.78)	59 (6.30)	
High school diploma/GED	78 (14.69)	135 (17.02)	166 (17.72)	
Vocational or training school	51 (9.60)	88 (11.10)	114 (12.17)	
Some college or associate degree	129 (24.29)	229 (28.88)	311 (33.19)	
College degree or baccalaureate degree	79 (14.88)	94 (11.85)	71 (7.58)	
Postgraduate degree	185 (34.84)	217 (27.36)	216 (23.05)	
Smoking				0.52
Never smokers	266 (50.96)	394 (49.68)	480 (51.28)	
Current smokers	39 97.47)	56 (7.06)	51 (5.45)	
Ever smokers	217 (41.57)	343 (43.25)	405 (43.27)	
Alcohol				<0.0001
Never drink	56 (10.63)	87 (10.94)	115 (12.29)	
<1 drink/month	48 (9.11)	106 (13.33)	170 (18.16)	
<1 drink/week	100 (18.98)	174 (21.89)	190 (20.30)	
1– <7 drinks/week	175 (33.21)	209 (26.29)	167 (17.84)	
≥7 drinks/week	87 (16.51)	94 (11.82)	66 (7.05)	
Past drinking	61 (11.57)	125 (15.72)	228 (24.36)	

Note: Removed 10 underweight participants from the analyses; removed missing group for race/ethnicity, family history, full term pregnancy, age at first full live birth, age at menarche, previous biopsy, not randomized to HRT, education, smoking and alcohol; abbreviations: BMI: Body mass index; SD: Standard deviation; HT: Hormone therapy; DM: Dietary modification; GED: General education development.

**Table 3:** Association of BMI and interval breast cancer diagnosed within 1 year after last normal mammogram screening compared to screening-detected breast cancers.

	Odds Ratio (95% Confidence Interval) ^1^					
	Model 1 ^2^	Model 2 ^3^	Model 3 ^4^	Model 4 ^5^	Model 5 ^6^	Model 6 ^7^
BMI Categories						
Underweight (BMI: <18.5)	1.16 (0.24, 5.53)	1.14 (0.24, 5.47)	1.15 (0.24, 5.49)	1.13 (0.24, 5.43)	0.98 (0.20, 4.81)	0.95 (0.19, 4.70)
Normal weight (BMI: 18.5–24.9)	1.00 (Ref)	1.00 (Ref)	1.00 (Ref)	1.00 (Ref)	1.00 (Ref)	1.00 (Ref)
Overweight (BMI: >24.9–29.9)	0.86 (0.64, 1.16)	0.88 (0.65, 1.198)	0.88 (0.66, 1.20)	0.89 (0.66, 1.21)	0.93 (0.69, 1.27)	0.91 (0.67, 1.24)
Obese (BMI: >29.9)	0.55 (0.41, 0.75)	0.57 (0.41, 0.80)	0.58 (0.42, 0.81)	0.60 (0.43, 0.84)	0.65 (0.46, 0.92)	0.62 (0.43, 0.89)
BMI Continuous Variables						
Per 1 unit increase	0.96 (0.94, 0.98)	0.96 (0.94, 0.98)	0.96 (0.94, 0.98)	0.96 (0.94, 0.99)	0.97 (0.94, 0.99)	0.97 (0.94, 0.99)

Note: ^1.^ This table reports a series of sequential multivariate models where a new variable is added to each model. Numbers in cells represent the odds ratios and 95% confidence intervals computed from the logistic regression model. ^2.^ Model 1: Unadjusted model. ^3.^ Model 2: Model 1 + WHR. ^4.^ Model 3: Model 2 + Gail 5 year risk score. ^5.^ Model 4: Model 3+ total dietary energy intake and total energy from recreational physical activity. ^6.^ Model 5: Model 4+ hormone replacement therapy clinical trial arm, dietary modification trial arm, smoking, alcohol intake, education and comorbidity. ^7.^ Model 6: Replace Model 5′s Gail 5-year risk with the original variables: age, ethnicity, age at menarche, age at first full term birth, family history of breast cancer, and previous breast biopsy. Abbreviation: BMI: Body mass index; WHR: Waist hip ratio.

**Table 4:** Association of BMI and interval breast cancer (IBC) diagnosed within 1 year after last normal mammogram screening compared to screening-detected breast cancers (SBC) sensitivity analyses.

Excluding Breast Cancer Cases Diagnosed within 2 Years (*n* = 285 for IBC and *n* = 1865 for SBC)						
	**Odds Ratio (95% Confidence Interval)** ^1^					
	Model 1 ^2^	Model 2 ^3^	Model 3 ^4^	Model 4 ^5^	Model 5 ^6^	Model 6 ^7^
BMI Categories						
Underweight (BMI: <18.5)	1.20 (0.25, 5.75)	1.20 (0.25, 5.73)	1.20 (0.25, 5.75)	1.19 (0.25, 5.70)	1.03 (0.21, 5.10)	0.98 (0.20, 4.87)
Normal weight (BMI: 18.5–24.9)	1.00 (Ref)	1.00 (Ref)	1.00 (Ref)	1.00 (Ref)	1.00 (Ref)	1.00 (Ref)
Overweight (BMI: >24.9–29.9)	0.80 (0.59, 1.10)	0.81 (0.59, 1.11)	0.81 (0.59, 1.12)	0.82 (0.59, 1.12)	0.85 (0.61, 1.18)	0.83 (0.60, 1.15)
Obese (BMI: >29.9)	0.54 (0.39, 0.74)	0.55 (0.39, 0.77)	0.55 (0.39, 0.78)	0.56 (0.39, 0.79)	0.60 (0.42, 0.87)	0.57 (0.39, 0.84)
BMI Continuous Variables						
Per 1 unit increase	0.96 (0.93, 0.98)	0.96 (0.93, 0.98)	0.96 (0.94, 0.98)	0.96 (0.94, 0.99)	0.97 (0.94, 0.99)	0.96 (0.94, 0.99)
**Excluding breast cancer cases diagnosed within 4 years (*n* = 212 for IBC and *n* = 1405 for SBC)**						
	**Odds Ratio (95% Confidence Interval)** ^1^					
	Model 1 ^2^	Model 2 ^3^	Model 3 ^4^	Model 4 ^5^	Model 5 ^6^	Model 6 ^7^
BMI Categories	Odds Ratio					
Underweight (BMI: <18.5)	1.55 (0.31, 7.85)	1.59 (0.31, 8.04)	1.61 (0.32, 8.18)	1.59 (0.31, 8.08)	1.28 (0.24, 6.88)	1.25 (0.23, 6.79)
Normal weight (BMI: 18.5–24.9)	1.00 (Ref)	1.00 (Ref)	1.00 (Ref)	1.00 (Ref)	1.00 (Ref)	1.00 (Ref)
Overweight (BMI: >24.9–29.9)	0.73 (0.51, 1.04)	0.69 (0.48, 1.01)	0.70 (0.48, 1.01)	0.70 (0.48, 1.02)	0.75 (0.51, 1.09)	0.73 (0.50, 1.08)
Obese (BMI: <29.9)	0.53 (0.37, 0.76)	0.49 (0.33, 0.72)	0.49 (0.33, 0.73)	0.49 (0.33, 0.74)	0.56 (0.37, 0.86)	0.55 (0.35, 0.85)
BMI Continuous Variables						
Per 1 unit increase	0.96 (0.94, 0.99)	0.96 (0.93, 0.99)	0.96 (0.93, 0.99)	0.96 (0.93, 0.99)	0.97 (0.94, 1.00)	0.97 (0.94, 1.00)
**Excluding breast cancer cases on E + P (*n* = 267 for IBC, *n* = 1628 for SBC)**						
	**Odds Ratio (95% Confidence Interval)** ^1^					
	Model 1 ^2^	Model 2 ^3^	Model 3 ^4^	Model 4 ^5^	Model 5 ^6^	Model 6 ^7^
BMI Categories						
Underweight (BMI: <18.5)	0.83 (0.10, 6.97)	0.82 (0.10, 6.92)	0.84 (0.10, 7.06)	0.82 (0.10, 6.91)	0.82 (0.10, 7.00)	0.70 (0.08, 6.05)
Normal weight (BMI: 18.5–24.9)	1.00 (Ref)	1.00 (Ref)	1.00 (Ref)	1.00 (Ref)	1.00 (Ref)	1.00 (Ref)
Overweight (BMI: >24.9–29.9)	0.96 (0.70, 1.33)	0.98 (0.70, 1.36)	0.98 (0.70, 1.37)	0.99 (0.71, 1.38)	1.04 (0.74, 1.46)	1.00 (0.71, 1.40)
Obese (BMI: <29.9)	0.60 (0.43, 0.84)	0.62 (0.43, 0.89)	0.62 (0.43, 0.90)	0.64 (0.44, 0.93)	0.68 (0.46, 1.01)	0.63 (0.42, 0.94)
BMI Continuous Variables						
Per 1 unit increase	0.96 (0.94, 0.98)	0.96 (0.94, 0.99)	0.96 (0.94, 0.99)	0.96 (0.93, 0.99)	0.97 (0.94, 0.995)	0.96 (0.94, 0.99)
**Including early-stage breast cancer (in situ + localized) (*n* = 218 for IBC, *n* = 1599 for SBC)**						
	**Odds Ratio (95% Confidence Interval)** ^1, 8^					
	Model 1 ^2^	Model 2 ^3^	Model 3 ^4^	Model 4 ^5^	Model 5 ^6^	Model 6 ^7^
BMI Categories						
Underweight (BMI: <18.5)	1.50 (0.31, 7.23)	1.45 (0.30, 6.99)	1.46 (0.30, 7.03)	1.47 (0.31, 7.11)	1.23 (0.24, 6.19)	1.20 (0.24, 6.11)
Normal weight (BMI: 18.5–24.9)	1.00 (Ref)	1.00 (Ref)	1.00 (Ref)	1.00 (Ref)	1.00 (Ref)	1.00 (Ref)
Overweight (BMI: >24.9–29.9)	1.03 (0.73, 1.46)	1.11 (0.78, 1.58)	1.11 (0.78, 1.59)	1.12 (0.78, 1.60)	1.18 (0.82, 1.71)	1.17 (0.81, 1.70)
Obese (BMI: >29.9)	0.54 (0.37, 0.78)	0.62 (0.41, 0.93)	0.62 (0.42, 0.94)	0.63 (0.42, 0.96)	0.71 (0.46, 1.09)	0.70 (0.45, 1.09)
BMI Continuous Variables						
Per 1 unit increase	0.95 (0.93, 0.98)	0.96 (0.94, 0.99)	0.96 (0.94, 0.99)	0.97 (0.94, 0.99)	0.97 (0.94, 1.00)	0.97 (0.94, 1.00)
**Including late-stage breast cancer (regional + distant) (*n* = 103 for IBC, *n* = 349 for SBC)**						
	**Odds Ratio (95% Confidence Interval)** ^1, 8^					
	Model 1 ^2^	Model 2 ^3^	Model 3 ^4^	Model 4 ^5^	Model 5 ^6^	Model 6 ^7^
BMI Categories						
Underweight (BMI: <18.5)	NA	NA	NA	NA	NA	NA
Normal weight (BMI: 18.5–24.9)	1.00 (Ref)	1.00 (Ref)	1.00 (Ref)	1.00 (Ref)	1.00 (Ref)	1.00 (Ref)
Overweight (BMI: >24.9–29.9)	0.51 (0.29, 0.93)	0.48 (0.26, 0.88)	0.47 (0.26, 0.87)	0.48 (0.26, 0.89)	0.54 (0.29, 1.02)	0.49 (0.25, 0.93)
Obese (BMI: >29.9)	0.51 (0.29, 0.87)	0.43 (0.23, 0.80)	0.45 (0.24, 0.84)	0.47 (0.25, 0.88)	0.54 (0.27, 1.08)	0.47 (0.22, 0.97)
BMI Continuous Variables						
Per 1 unit increase	0.95 (0.91, 0.99)	0.94 (0.90, 0.98)	0.94 (0.90, 0.99)	0.95 (0.90, 0.99)	0.95 (0.91, 1.00)	0.95 (0.90, 1.00)

^1^. This table reports a series of sequential multivariate models where a new variable is added to each model. Numbers in cells represent the odds ratios and 95% confidence intervals computed from the logistic regression model. ^2^. Model 1: Unadjusted model. ^3^. Model 2: Model 1 + WHR. ^4.^ Model 3: Model 2 + Gail 5 year risk score. ^5^. Model 4: Model 3+ total dietary energy intake and total energy from recreational physical activity. ^6^. Model 5: Model 4+ for hormone replacement therapy clinical trial arm and dietary modification trial arm, smoking status, alcohol intake, education and comorbidity. ^7^. Model 6: Replace Gail 5-year risk with the original variables: age, ethnicity, age at menarche, age at first full term birth, family history of breast cancer, previous breast biopsy. ^8^. Among the 2293 participants included in the analyses, we had 24 missing stage pieces of information; other numbers by stage are: in-situ (*n* = 443), localized (*n* = 1374), regional (*n* = 429), distant (*n* = 23).

## Data Availability

Publicly available (upon approval) datasets were analyzed in this study. This data can be found here: https://www.whi.org/page/working-with-whi-data (accessed on 10 April 2020).

## Supplementary Materials

The following supporting information can be downloaded at: https://www.mdpi.com/article/10.3390/cancers14133228/s1, Supplemental Table S1: Association of BMI (categorical variable) and interval breast cancer diagnosed within 1 year after last normal mammogram screening compared to screening-detected breast cancers. Supplemental Table S2: Association of BMI (continuous variable) and interval breast cancer diagnosed within 1 year after last normal mammogram screening compared to screening-detected breast cancers. Supplemental Table S3: Association of waist-to-hip ratio (WHR) and interval breast cancer diagnosed within 1 year after last normal mammogram screening compared to screening-detected breast cancers. Supplemental Table S4: Mammogram screening adherence of trial cohorts and included or non-compliant participants.
