# Supplementary material for: Body Mass Index Is Inversely Associated with Risk of Postmenopausal Interval Breast Cancer: Results from the Women’s Health Initiative
Source: Cancers (Basel). 2022 Jun 30;14(13):3228. doi: 10.3390/cancers14133228 (PMC9264843; doi:10.3390/cancers14133228)
Supplement: Supplementary file 1 [file cancers-14-03228-s001.zip › cancers-1779916-supplementary.pdf]

cancers

MDPI

Table S1. Association of BMI (categorical variable) and interval breast cancer diagnosed within 1 year after last normal mammogram screening compared to screening-detected breast cancers .

	Odds Ratio					
	Model 1	Model 2	Model 3	Model 4	Model 5[6]	Model 6[7]
**BMI Categories**						
Underweight (BMI <18.5)	1.16 (0.24, 5.53)	1.14 (0.24, 5.47)	1.15 (0.24, 5.49)	1.13 (0.24, 5.43)	0.98 (0.20, 4.81)	0.95 (0.19, 4.70)
Normal weight (BMI:18.5-24.9)	1.00 (Ref)	1.00 (Ref)	1.00 (Ref)	1.00 (Ref)	1.00 (Ref)	1.00 (Ref)
Overweight (BMI: >24.9-29.9)	0.86 (0.64, 1.16)	0.88 (0.65, 1.198)	0.88 (0.66, 1.20)	0.89 (0.66, 1.21)	0.93 (0.69, 1.27)	0.91 (0.67, 1.24)
Obese (BMI > 29.9)	**0.55 (0.41, 0.75)**	**0.57 (0.41, 0.80)**	**0.58 (0.42, 0.81)**	**0.60 (0.43, 0.84)**	**0.65 (0.46, 0.92)**	**0.62 (0.43, 0.89)**
**Covariates**						
Waist-to-hip ratio		0.57 (0.10, 3.31)	0.54 (0.09, 3.18)	0.53 (0.09, 3.13)	0.52 (0.09, 3.15)	0.60 (0.10, 3.72)
Gail 5-year risk			**1.11 (1.00, 1.22)**	**1.11 (1.00, 1.22)**	**1.11 (1.00, 1.22)**	**n/a**
Total Energy intake				1.00 (1.00, 1.00)	1.00 (1.00, 1.00)	1.00 (1.00, 1.00)
Total energy expended from recreational physical activity (MET-hours/week)				1.00 (0.99, 1.01)	1.00 (0.99, 1.02)	1.00 (0.99, 1.02)
Comorbidity					1.00 (Ref)	1.00 (Ref)
0					1.05 (0.77, 1.42)	1.07 (0.78, 1.45)
1					1.12 (0.72, 1.85)	1.17 (0.72, 1.90)
2					2.09 (1.09, 4.00)	2.27 (1.17, 4.04)
≥3						
Age at screening						1.00 (0.98, 1.02)
Race/ethnicity						
White						1.00 (Ref)
African American						0.96 (0.59, 1.59)
Hispanic						2.17 (1.13, 4.17)
Asian						0.85 (0.38, 1.89)
Family history of breast cancer						
No						1.00 (Ref)
Yes						1.18 (0.89, 1.57)
Age at first live birth						
Never had any live birth						0.99 (0.45,2.19)
<20 years old						0.68 (0.43, 1.06)
20-29 years old						1.00 (Ref)
≥30 years old						1.24 (0.82, 1.90)
Age at menarche, No (%)						
≤13 years old						1.00 (Ref)
>13 years old						**0.71 (0.52, 0.97)**
Previous breast biopsy, No (%)						
0						1.00 (Ref)
1						1.16 (0.85, 1.58)
>1						1.39 (0.87, 2.20)

This table reports a series of sequential multivariate models where a new variable is added to each model. Numbers in cells represent the Odds Ratios and 95% confidence intervals computed from the Logistic Regression model. Model 1: Unadjusted model; Model 2: Model 1 + WHR; Model 3: Model 2 + Gail 5 year risk score; Model 4: Model 3+ total dietary energy intake and total energy from recreational physical activity; Model 5: Model 4+ for hormone replacement therapy clinical trial arm and dietary modification trial arm, smoking status, alcohol intake, education and comorbidity; Model 6: Replace Gail 5-year risk with the original variables: age, ethnicity, age at menarche, age at first full term birth, family history of breast cancer, previous breast biopsy.

Table S2. Association of BMI (continuous variable) and interval breast cancer diagnosed within 1 year after last normal mammogram screening compared to screening-detected breast cancers.

	Odds Ratio					
	Model 1	Model 2	Model 3	Model 4	Model 5[6]	Model 6[7]
**BMI Continuous Variables**						
Per 1 unite increase	**0.96 (0.94, 0.98)**	**0.96 (0.94, 0.98)**	**0.96 (0.94, 0.98)**	**0.96 (0.94, 0.99)**	**0.97 (0.94, 0.99)**	**0.97 (0.94, 0.99)**
**Covariates**						
Waist-to-hip ratio		0.57 (0.10, 3.27)	0.54 (0.09, 3.13)	0.53 (0.09, 3.08)	0.52 (0.09, 3.11)	0.57 (0.09, 3.47)
Gail 5-year risk			**1.10 (1.00, 1.22)**	**1.11 (1.00, 1.22)**	**1.11 (1.00, 1.22)**	**n/a**
Total Energy intake				1.00 (1.00, 1.00)	1.00 (1.00, 1.00)	1.00 (1.00, 1.00)
Total energy expended from recreational physical activity (MET-hours/week)				1.00 (0.99, 1.01)	1.00 (0.99, 1.02)	1.00 (0.99, 1.02)
Comorbidity					1.00 (Ref)	1.00 (Ref)
0					1.05 (0.77, 1.42)	1.07 (0.78, 1.45)
1					1.11 (0.69, 1.79)	1.15 (0.71, 1.86)
2					2.11 (1.10, 4.04)	2.33 (1.20, 4.52)
≥3						
Age at screening						1.00 (0.98, 1.02)
Race/ethnicity						
White						1.00 (Ref)
African American						0.96 (0.58, 1.57)
Hispanic						2.26 (1.18, 4.34)
Asian						0.82 (0.37, 1.83)
Family history of breast cancer						
No						1.00 (Ref)
Yes						1.17 (0.88, 1.56)
Age at first live birth						
Never had any live birth						0.96 (0.44,2.12)
<20 years old						0.69 (0.44, 1.08)
20-29 years old						1.00 (Ref)
≥30 years old						1.25 (0.82, 1.90)
Age at menarche REDO, No (%)						
≤13 years old						1.00 (Ref)
>13 years old						**0.72 (0.53, 0.98)**
Previous breast biopsy, No (%)						
0						1.00 (Ref)
1						1.15 (0.84, 1.57)
>1						1.37 (0.86, 2.18)

This table reports a series of sequential multivariate models where a new variable is added to each model. Numbers in cells represent the Odds Ratios and 95% confidence intervals computed from the Logistic Regression model. Model 1: Unadjusted model; Model 2: Model 1 + WHR; Model 3: Model 2 + Gail 5 year risk score; Model 4: Model 3+ total dietary energy intake and total energy from recreational physical activity; Model 5: Model 4+ for hormone replacement therapy clinical trial arm and dietary modification trial arm, smoking status, alcohol intake, education and comorbidity; Model 6: Replace Gail 5-year risk with the original variables: age, ethnicity, age at menarche, age at first full term birth, family history of breast cancer, previous breast biopsy.

Table S3. Association of waist-to-hip ratio (WHR) and interval breast cancer diagnosed within 1 year after last normal mammogram screening compared to screening-detected breast cancers .

	Odds Ratio (95% Confidence Interval)[1]				
	Model 1[2]	Model 2[3]	Model 3[4]	Model 4[5]	Model 5[6]
**WHR Continuous Variables**					
Per 1 unite increase	**0.16 (0.03, 0.84)**	**0.16 (0.03, 0.84)**	**0.17 (0.03, 0.90)**	0.22 (0.04, 1.18)	0.23 (0.04, 1.27)

This table reports a series of sequential multivariate models where a new variable is added to each model. Numbers in cells represent the Odds Ratios and 95% confidence intervals computed from the Logistic Regression model. Model 1: Unadjusted model; Model 2: Model 1 + Gail 5 year risk score; Model 3: Model 2 + total dietary energy intake and total energy from recreational physical activity; Model 4: Model 3+ hormone replacement therapy clinical trial arm, dietary modification trial arm, smoking, alcohol intake, education and comorbidity; Model 5: Replace Model 4's Gail 5-year risk with the original variables: age, ethnicity, age at menarche, age at first full term birth, family history of breast cancer, and previous breast biopsy.

Table S4. Mammogram screening adherence of trial cohorts and included or non-compliance participants.

	**Included IBC< 1 yr (N=324)**	**Non-compliance (N=2,477)**	**P value**
**Mean (SD) of BMI** continuous variable	28.09 (5.36)	29.52 (5.96)	**<0.0001**
**Total number of mammograms** before diagnosis	4.33 (2.62)	6.17 (2.82)	<0.0001
**HT Study group[d], No. (%)**			0.0003
Estrogen-alone intervention	20 (6.17)	126 (5.09)	
Estrogen-alone control	21 (6.48)	134 (5.41)	
Estrogen + progestin intervention	57 (17.59)	251 (10.13)	
Estrogen + progestin control	33 (10.19)	220 (8.88)	
Not randomized to HT	193 (59.57)	1746 (70.49)	
**DM Trial group[e], No. (%)**			0.003
Intervention	92 (28.40)	777 (31.37)	
Control	140 (43.21)	1200 (48.45)	
Not randomized to DM	92 (28.40)	500 (20.19)	
